# Automatic discontinuation of isolation precautions and electronic alerts of MDRO positive patients: safe or sorry?

**DOI:** 10.1186/2047-2994-4-S1-P119

**Published:** 2015-06-16

**Authors:** M Bos, A Voss, A Tostmann, J Hopman

**Affiliations:** 1 Medical Microbiology/Hygiene and Infection Prevention, Radboud University Medical Centre Nijmegen, Nijmegen, Netherlands

## Introduction

The Netherlands have a low prevalence of Multi drug Resistant organisms (MDRO), in part due to their national guideline concerning MDRO carriers. Apart from being flagged in Electronic Health Records, immediate isolation precautions must be taken if the last MDRO positive culture is less than 1 year ago. No clear guidance is given on duration and termination of electronic alerts and isolation precautions for MDRO positive patients.

## Objectives

To determine the proportion of persistent MDRO carriage in patients who were screened in 2014.

## Methods

We performed a retrospective study in our tertiary care university medical centre by reviewing the data of all patients with MDRO alerts who were screened in 2014 to determine if MDRO carriage was still present. Patients with MRSA/VRE/CRE carriage were excluded, since they follow different guidelines.

Patients who are carrier of MDRO are cultured twice (throat and perianal swab) at least 24 hours apart. After 2 negative screening results of both sites, the MDRO carriage is considered not to be present anymore.

## Results

In 2014, 238 patients were cultured for MDRO carriage. Median age was 59 (IQR 29 -70) years, 55% (n=130) was male. Of the initial MDRO positive cultures, the most common material were urine (53%), rectal/perianal/faecal (23%) and sputum cultures (6%). Most common micro-organism was Escherichia coli (n=136) with ESBL as the most prevalent resistance mechanism (35%).The median time between initial positive MDRO culture and control cultures was 499 days (IQR 187-1055)

Fifty-six patients tested MDRO positive in the first control cultures. Sixty-seven patients were screened a second time, of which 21 were MDRO positive (after initial negative cultures). 48% (95% CI 37.4-71.6) of the patients with the initial MDRO positive culture < 1 year ago, remained MDRO positive.

Of the patients who had been labelled MDRO positive longer than 1 year ago, 26% (95% CI 20.2-34.6%) remained MDRO positive.

## Conclusion

Twenty-six percent of the patients labelled MDRO positive longer than 1 year ago, remain MDRO positive and can be a source of MDRO transmission within the hospital. Discontinuation of electronic alerts and isolation precautions for MDRO carriage should therefore be based on microbiological screening results.

## Disclosure of interest

None declared.

